# Dietary Cholesterol Affects Plasma Lipid Levels, the Intravascular Processing of Lipoproteins and Reverse Cholesterol Transport without Increasing the Risk for Heart Disease 

**DOI:** 10.3390/nu4081015

**Published:** 2012-08-17

**Authors:** Jacqueline Barona, Maria Luz Fernandez

**Affiliations:** 1 Department of Nutritional Sciences, University of Connecticut, Storrs, CT 06269, USA; Email: Jacqueline.barona@uconn.edu; 2 School of Microbiology, University of Antioquia, Medellin, A.A. 1226, Colombia

**Keywords:** dietary cholesterol, lipoprotein metabolism, LDL/HDL ratio, clinical interventions, epidemiological studies

## Abstract

The associations between dietary cholesterol and heart disease are highly controversial. While epidemiological studies and clinical interventions have shown the lack of correlation between cholesterol intake and cardiovascular disease (CVD) risk, there is still concern among health practitioners and the general population regarding dietary cholesterol. In this review, several clinical studies utilizing cholesterol challenges are analyzed in terms of changes that occur in lipoprotein metabolism resulting from excess consumption of cholesterol. Dietary cholesterol has been shown to increase both LDL and HDL in those individuals who respond to a cholesterol challenge without altering the LDL cholesterol/HDL cholesterol ratio, a key marker of CVD risk. Further, dietary cholesterol has been shown to increase only HDL with no changes in LDL with average cholesterol consumption and during weight loss interventions. Ingestion of cholesterol has also been shown to increase the size of both LDL and HDL particles with the associated implications of a less atherogenic LDL particle as well as more functional HDL in reverse cholesterol transport. Other changes observed in lipoprotein metabolism are a greater number of large LDL and decreases in small LDL subfractions. All this information put together points to specific roles of dietary cholesterol in substantially altering intravascular processing of lipoproteins as well as reverse cholesterol transport.

## 1. Introduction

Elevated levels of plasma LDL cholesterol (LDL-C) are associated with increased risk of cardiovascular disease (CVD) [1]. Thus dietary interventions that lower LDL are foremost in the mind of health practitioners. However, it is important to remember that low levels of HDL cholesterol such as we observe in the case of metabolic syndrome (MetS) and in diabetic individuals also represent a well established risk for the development of CVD [2]. Although it is still not part of the NCEP/ATP guidelines, the balance between LDL and HDL is considered a key marker of CVD risk making the LDL-C/HDL cholesterol ratio a valuable tool for the assessment and clinical management of individuals at risk for heart disease [3].

Dietary cholesterol is a highly controversial nutrient because it raises plasma cholesterol, especially in those individuals who are not able to maintain plasma cholesterol homeostasis by decreasing absorption in the small intestine or by suppressing synthesis [4]. It is important to understand that these individuals classified as hyper-responders to dietary cholesterol do not constitute the whole population (about 25%) and that the majority of people have a normal response to dietary cholesterol [5]. 

## 2. Dietary Cholesterol and Plasma LDL and HDL

Studies conducted in children [6], young women and men [7,8] and elderly individuals [9] who were assigned to consume additional 550 mg of cholesterol per day in the case of children and additional 640 mg of cholesterol in the case of adults for 4 weeks have shown that hyper-responders raise both LDL and HDL thus the LDL/HDL ratio is maintained. For those individuals that are not hyper-responders, the LDL/HDL is also maintained since no significant increases in plasma cholesterol are observed [6,7,8,9]. However, during weight loss interventions [10,11], only HDL cholesterol is increased substantially while LDL levels are either maintained or there are non-significant increases. When individuals consume between 180 and 230 additional mg of dietary cholesterol (one egg per day), only HDL is raised [12] All these studies are considered dietary cholesterol challenges based on the dietary guidelines for Americans 2010 that recommend 300 mg/day for healthy populations and 200 mg/day for individuals with increased risk for CVD [13].

Contrary to the belief that eggs and dietary cholesterol should not be provided to patients at risk for CVD [14], effects of cholesterol intake, in individuals classified with MetS, have not shown any detrimental effect on lipoprotein profiles [15]. MetS is characterized by central obesity, elevated blood pressure, high plasma fasting glucose and dyslipidemias (elevated triglycerides (TG) and low HDL). Studies conducted in individuals with MetS have demonstrated that consuming additional 550 mg of dietary cholesterol (via eggs) for 12 weeks results in a very significant increase in HDL (*p* < 0.0001) with no changes in LDL [15]. This is a very significant finding considering that low HDL is one of the features of MetS that predisposes for heart disease risk. 

## 3. Dietary Cholesterol and LDL

It is well established that LDL particle size plays a major role in the atherogenicity of this particle [16]. Thus individuals have been classified as pattern A if the large, buoyant LDL particles are the more prominent particles [17]. In contrast, pattern B is characterized by higher concentrations of small, dense LDL, making individuals with this classification have three times greater risk for CVD [18]. Small LDL possess a number of features that make these particles more atherogenic, including their ability to penetrate the arterial wall and become easily oxidized, making them a perfect target for macrophages [18]. Macrophages can then become foam cells and initiate the whole process of atherosclerosis [19].

Dietary cholesterol has been shown to increase LDL size in numerous studies [20,21,22]. In addition to size, cholesterol also alters the number of large and small LDL particles [20,21]. Dietary cholesterol substantially modifies lipoprotein subpopulations and size. In the context of a low carbohydrate diet, a challenge of additional 640 mg of cholesterol per day, results in larger LDL diameter [23], higher concentrations of the large LDL particle and reduced number of the small atherogenic LDL. Other studies have also reported increases in LDL cholesterol as a result of a cholesterol challenge [5,24]. However, a consistent increase in HDL cholesterol has also been observed [10,11,12,24]. Further, increases in LDL size and in the large LDL subfractions are regularly reported [20,23,25]. These lipoprotein profiles resulting from a dietary cholesterol challenge do not appear to be related to increased risk for CVD.

It is important to mention that LDL cholesterol does not increase under all circumstances or in all cases following a cholesterol challenge. When the individual response is analyzed, a proportion of about 2/3 of individuals do not experience increases in LDL cholesterol [11,26] even after a challenge of 640 mg/day per day for 4 weeks [7,8,9]. In contrast, a consistent finding includes decreases in small LDL subfractions [22,23], increases in large LDL [14,21] and increases in LDL size [6,20,23] with some individuals shifting from the more atherogenic pattern B to pattern A [6,23] after consuming dietary cholesterol.

## 4. Dietary Cholesterol and HDL

According to the nuclear magnetic resonance (NMR) spectroscopy method, HDL subclasses can be grouped as large HDL, 8.8–13 nm; medium HDL, 8.2–8.8 nm; and small HDL particles 7.3–8.2 nm [27]. Large HDL measured by NMR has been negatively associated with incident CVD (*p* < 0.001) [28,29]; and this particle has been shown to decrease in the case of coronary artery disease in patients with Type 1 diabetes [30]. We have observed that the increases in HDL cholesterol are associated with increases in the number of large HDL particles [23]. Additionally, it has been observed that HDL particle size increased independent of response classification (hyper- or hypo-responders) to dietary cholesterol only in those consuming 3 eggs/day for 4 weeks, compared with egg substitute [22]. However, the concentration of the large HDL was greater in hyper-responders compared to hypo-responders [22]. 

Several studies in overweight/obese, normo- and hypercholesterolemic individuals [22,23,31] have shown that excess dietary cholesterol intake promotes the formation of the larger HDL particle by becoming enriched in cholesterol [32]. In contrast, in a crossover study, cholesterol supplementation of 800 mg/day during 3 weeks did not result in significant changes on the concentration of HDL subclasses, isolated by density gradient ultracentrifugation, or total HDL mass in insulin-dependent diabetic patients (with normal lipid values) compared with placebo [33]. Conversely, in normal control participants this concentration of dietary cholesterol resulted in a significant increase in the mean concentration of the large HDL2a by 12.2% [33]. This change was significantly different from that observed in the diabetic patients, in whom a similar, but non-significant increase in the mean concentration of HDL2b was observed [33]. Similarly, the relative cholesterol content in HDL subclasses significantly increased after cholesterol supplementation only in the control volunteers while the relative content of TG tended to decrease in all HDL subclasses with cholesterol supplementation, reaching significance for the large HDL2b subclass and total HDL [33].

Besides the proposed genetic influence on the responses (hyper- or hypo-responders) to dietary cholesterol [26,34] and consequently on HDL, there are other factors that may be related to the increases in HDL after excess cholesterol intake. In a crossover, randomized, double-blind study [35], 26 men and 25 women were matched for age, LDL cholesterol, TG, and body mass index (BMI), to consume in a random order, two isocaloric supplements containing either 650 mg cholesterol plus 31 g fat/day or fat/cholesterol free each for 3 weeks, after a baseline low fat diet. Although at baseline HDL2 cholesterol was significantly higher in women than in men, women showed greater increases (21.5%) of HDL2 cholesterol than men (11%) in response to the fat and cholesterol supplement [35]. The authors suggested that women are more efficient than men at incorporating dietary cholesterol into the large HDL2 by enhanced transfer of free cholesterol to HDL from triglyceride-rich particles during the delipidation cascade [35]. In addition to gender, age and BMI influenced the response to fat/cholesterol supplementation in men, with the older men (≥50 years) and men with BMI > 25 kg/m^2^ showing no rise in HDL2 cholesterol [35]. Similar results were found in a further study evaluating 120 men and women, in which the authors indicated that the different response to dietary cholesterol challenges between men and women can be partly explained by their differences in fat distribution [36]. 

In summary, the majority of the studies presented in this review have reported raises in HDL-C and HDL size in response to dietary cholesterol challenges [15,22,23].

## 5. Dietary Cholesterol and Reverse Cholesterol Transport

Reports have indicated that the body responds to cholesterol challenges by increasing different steps involved in reverse cholesterol transport (RCT) [37,38]. RCT is the process involved in the uptake of cholesterol from peripheral tissues including macrophages and its return to the liver where it is targeted for biliary secretion [39]. 

A study conducted in 26 healthy men and women aged 20–57 years reported a significant decrease in newly synthesized cholesteryl ester as measured by cholesteryl ester transfer activity after consuming one additional egg/day over 12 days in women [40]. This study was of short duration (12 days), with small sample size to compare between genders; subjects were not matched with the controls, and were evaluated after consuming 240 mg cholesterol per day. Contrarily, later studies [7,20] have reported significant increases in CETP activity evaluating also healthy premenopausal women in response to 640 mg cholesterol intake per day during 30 days. In fact, Herron *et al*. [20] reported that CETP activity was significantly higher in women (*n* = 27) than in men (*n* = 25) after the cholesterol challenge period. 

It has been suggested that the relative concentration of HDL subclasses and the increases in RCT, are more important than HDL-cholesterol *per se*, which determine the potential antiatherogenic outcomes [41,42]. CETP facilitates the equimolar exchange of cholesteryl esters (CE) from HDL for TG in apoB100-containing lipoproteins [39]. Thus CETP plays an active role in HDL remodeling [41]. Increases in CETP activity promotes the enrichment of apoB100-containing lipoproteins with CE, and it has been related to a decrease in HDL-C levels; thus, it is considered to be pro-atherogenic [7,43]. However, when the increases of CETP activity are not associated with reduction in HDL-C levels, as seen in the above studies [7,20], this molecule is considered as anti-atherogenic by enhancing RCT through the CE-enrichment of LDL, which can be taken up by the liver, where CE are metabolized [7]. Similarly, when the raises of HDL-C levels are related to greater synthesis (not less catabolism), beneficial effects on the prevention of CVD are observed [42]. 

Additionally, the promotion of macrophage RCT has been suggested as a potential therapeutic approach to prevent or reverse atherosclerotic vascular disease [33]. A study in mice provided evidence of the crucial role of dietary cholesterol signaling through liver ATP-binding cassette (ABC) transporters G5/G8 (ABCG5/G8) upregulation in the induction of macrophage-specific RCT [44]. Moreover, in the context of carbohydrate restriction and weight loss, we have shown that cholesterol efflux from macrophages incubated with the serum of individuals consuming 550 mg of cholesterol per day for 12 weeks is increased, possibly suggesting enhanced RCT [45].

Therefore, the increased concentration of the large HDL subclass, as pointed out before, in CETP activity and in RCT in the above studies may indicate potential antiatherogenic outcomes with excess dietary cholesterol/egg consumption.

**Figure 1:**
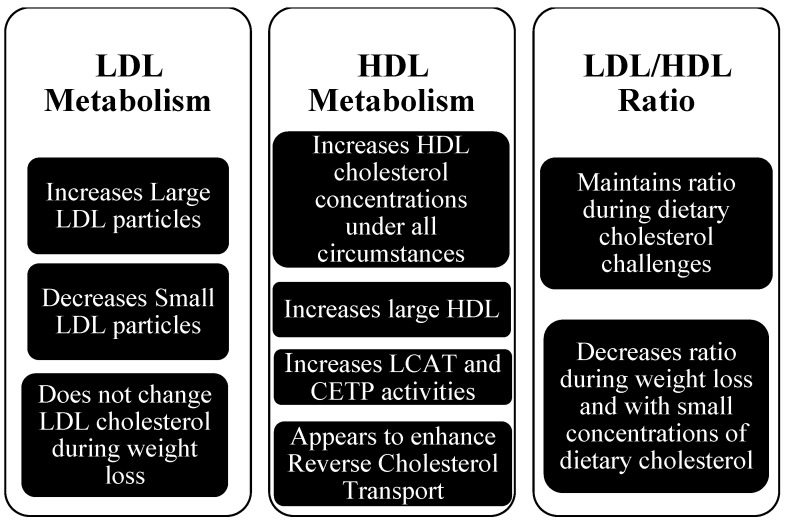
Effects of Dietary Cholesterol on LDL metabolism [5,6,7,8,9,10,11,12,23] HDL metabolism [5,6,7,8,9,10,11,12,23] and the LDL/HDL ratio [6,7,9,12,23].

A summary of the effects of dietary cholesterol on LDL, HDL metabolism and LDL/HDL ratio is presented in Figure 1.

## 6. Compensatory Mechanisms: Cholesterol Absorption and Cholesterol Synthesis

It is clearly established that the absorption of cholesterol depends on several factors including specific polymorphisms that may affect cholesterol transporters in the ileum [46], other dietary factors including plant sterols [47] and soluble fiber [48] and the amount of cholesterol consumed [49]. On average the absorption of dietary cholesterol is about 60% [50]. Human studies have demonstrated that individuals reduce absorption to maintain plasma cholesterol [51]. An interesting observation was the study by Kern F. [52] that reported that one individual who consumed 25 egg per day did not have increases in LDL cholesterol due to reduced absorption [52]. One study reported that only suppression of cholesterol synthesis was necessary to maintain plasma cholesterol [51].

The ABCG5/G8 may function to regulate the efflux of dietary sterols from intestinal epithelial cells back into the intestinal lumen and from the liver to the bile duct [53]. Therefore, these transporters play an important role in reducing intestinal absorption and promote biliary excretion of sterols [53]. In response to a high cholesterol diet, mice were shown to increase the expression of ABCG5/G8 [54]. Thus, polymorphisms in the genes that codify for these transporters may have an impact in the response to dietary cholesterol and cholesterol metabolism [55]. For example, Herron *et al.* [55] evaluated ABCG5 polymorphisms in 91 men and women who consumed 640 mg cholesterol provided by eggs or egg substitute during 30 days in a randomized crossover design study. Regardless of phenotype and gender, participants significantly increased their plasma TC and LDL-C concentrations during the egg period compared with egg substitute. However, volunteers with the ABCG5 C/C allele had significantly higher TC concentrations after egg consumption than those with the ABCG5 G (C/G and G/G combined) [55].

HMG-CoA reductase, the regulatory enzyme of cholesterol synthesis has been targeted by drug companies [56,57], mainly for its key role in maintaining plasma LDL cholesterol concentrations [58]. It is therefore not surprising that one of the compensatory mechanisms to maintain plasma LDL is targeted at this enzyme. The body has the ability to reduce synthesis as a response to dietary cholesterol challenges [59]. In animal studies, reductions of hepatic HMG-CoA reductase have been observed [60,61] as a response to excess dietary cholesterol. Similar responses have been observed in human studies in peripheral mononuclear cells [62,63]. Mutungi *et al.* [62] following a dietary cholesterol challenge, reported decreases in the expression of HMG-CoA reductase, as a compensatory mechanism to maintain plasma LDL cholesterol concentrations. 

In the context of carbohydrate restriction and weight loss, dietary cholesterol has been shown to modify the expression of genes regulating cholesterol homeostasis [62]. Both the expression of HMG-CoA reductase and the LDL receptor were down-regulated to maintain hepatic cholesterol homeostasis [54]. 

## 7. Conclusions

Dietary cholesterol promotes the formation of larger LDL [20,21,22,23] and HDL [15,23,45]. It has also been shown to increase LCAT [8,15] and CETP [7,20] activities, and promote cholesterol efflux from macrophages [45] indicating an enhancement of RCT. All this information taken together emphasizes the lack of correlation between dietary cholesterol and heart disease as it has been pointed out in multiple epidemiological studies [64,65,66,67].
